# Basic Principles for Setting MRLs for Pesticides in Food Commodities in Japan

**DOI:** 10.14252/foodsafetyfscj.D-23-00011

**Published:** 2024-06-06

**Authors:** Hiroshi Akiyama, Yusuke Iwasaki, Rie Ito

**Affiliations:** 1 Department of Analytical Chemistry, School of Pharmacy and Pharmaceutical Sciences, Hoshi University, 2-4-41 Ebara, Shinagawa-ku, Tokyo 142-0063, Japan; 2 Division of Foods, National Institute of Health Sciences, 3-25-26 Tonomachi, Kawasaki-ku, Kawasaki, Kanagawa 210-0821, Japan

**Keywords:** basic principle, food commodity, Japan, maximum residue limits, residual pesticides

## Abstract

The Committee on Pesticides and Veterinary Drugs of the Food Sanitation Council under the Pharmaceutical Affairs and Food Sanitation Council set the maximum residue limits (MRLs) for residual pesticides, veterinary drugs, and feed additives in food commodities according to the basic principles for establishing MRLs for pesticides in food commodities in Japan. The basic principles consist of the following seven concepts: 1. Outline of setting Japanese MRLs for pesticide residue in food commodities; 2. Preparation of draft MRLs for pesticides in livestock commodities; 3. Preparation of draft MRLs for pesticides in fish and shellfish; 4. Technical guideline for setting MRLs for pesticides, etc., in honey; 5. Methods of setting standards for chemical substances used as pesticides in the past that are now detected as contaminants; 6. Concept of setting MRLs for pesticides at an extremely low level; and 7. Commodity groups and representative commodities regarding MRLs based on international harmonization. The present paper introduces and explains the basic principles for establishing MRLs for pesticides, veterinary drugs, and feed additives in food commodities.

## Introduction

Food safety legislation in Japan is based on the 2003 “Food Safety Basic Law” and the 1947 “Food Sanitation Law”, which were enacted to protect public health. The Japanese government has the duty of formulating and enforcing comprehensive measures for ensuring food safety. The overall objective of the Food Safety Basic Law is to mandate measures for ensuring food safety. It defines the basic framework for ensuring food safety and the responsibilities of the national and local governments and food industry members, identifies the role of the consumer, and sets the basic policies for formulating specific measures based on risk analyses.

Risk analyses consist of risk assessment, risk management, and risk communication. As a responsible risk analysis agency within the Food Safety Commission of Japan (FSCJ) assesses the risks associated with residual pesticides, veterinary drugs, and feed additives in food commodities and recommends acceptable daily intakes (ADIs) and acute reference doses (ARfDs). As the agencies responsible for risk management, the Ministry of Health, Labour and Welfare (MHLW), the Ministry of Agriculture, Forestry and Fisheries (MAFF), and the Consumer Affairs Agency establish maximum residue limits (MRLs) and other standards related to risk management under the Food Sanitation Act, Agricultural Chemicals Regulation Act, and Food Labeling Act, respectively. All these agencies communicate risk in the form of scientific advice.

The Codex Alimentarius Commission (CAC) has employed a method under the Food and Agriculture Organization (FAO)/World Health Organization (WHO) Joint Meeting on Pesticide Residues (JMPR) in 1963 to monitor MRLs legally tolerated in food commodities with the established values expressed as mg/kg^1^^)^.

MRLs for pesticides in commodities have been set based on the concept of “Setting of the MRL values of pesticides in food commodities” (January 27, 2010, Committee on Pesticides and Veterinary Drugs of the Food Sanitation Council under the Pharmaceutical Affairs and Food Sanitation Council) in Japan. However, based on current international agreements and concepts, this concept for setting MRLs was completely revised, and basic principles for establishing or revising MRLs for pesticides in food commodities have been provided by the Committee on Pesticides and Veterinary Drugs of the Food Sanitation Council under the Pharmaceutical Affairs and Food Sanitation Council (hereafter “the Committee”) in 2019 as part of the MHLW^2^^)^ (first revision in March 2021, second revision in March 2023). The data requirements for setting MRLs are based as much as possible on the Organisation for Economic Co-operation and Development (OECD) guidelines, but also reflect relevant domestic guidelines. This paper introduces and explains the basic principles for establishing MRLs for pesticides, veterinary drugs, and feed additives in food commodities in Japan (revised in March, 2023)^2^^)^.

In addition, regarding the formulation of standards for food products (e.g., pesticide residue standards) and other matters related to the administration of food hygiene standards, to ensure food safety based on scientific knowledge, information on the comprehensive development of the environment necessary shall also be provided. The authority will be transferred from the MHLW to the Prime Minister (Consumer Affairs Agency) to integrate the affairs related to general coordination of matters, etc. in Japan (effective date: April 1, 2024).

## 1. Outline Regarding Setting Japanese MRLs for Pesticides in Food Commodities

### 1-1. Basic Concept for Setting Japanese MRLs for Pesticides in Food Commodities

To set and inspect conformity to the Japanese MRLs, the Committee should determine the appropriate definition of pesticide residue while referring to the MRLs established by the CAC (Codex MRLs). The Committee should prepare draft MRLs based on the Codex MRLs and results from supervised residue trials.

The Committee should also estimate the probable long- and short-term dietary intakes when the draft MRLs are adopted and confirm that these do not exceed the ADIs and ARfDs specified by the risk assessment performed by the FSCJ. The MRLs are determined in the case that the accumulated values of the estimated long-term dietary intakes do not exceed 80% of the theoretical maximum daily intake (TMDI) or estimated daily intake, and in the case that the estimated short-term dietary intakes do not exceed the ARfD.

When the Committee prepares draft MRLs, they basically adopt Codex MRLs for commodities for which they have been set. However, the residue levels in commodities should fluctuate as a result of 1) standards for use of pesticides (items described on the agricultural chemical labels, e.g., application method, pre-harvest interval [PHI]), 2) cultivation conditions (e.g., facility/open field, planting density), 3) climate (e.g., rainfall, sunshine, temperature), and 4) varieties (e.g., differences in crop sizes, forms, and leaf density).

Considering these fluctuations, the draft MRLs should be set based on data from supervised residue trials in the case that the residue levels exceeding the Codex MRLs are assumed in food commodities produced in Japan based on data from domestic supervised trials. In addition, in the case that the MRLs in other countries are higher than the Codex MRLs and evidence of the proposal MRLs is submitted in the form of data from supervised residue trials, the draft MRLs should be prepared considering the results of residue trials.

When the Committee sets MRLs for commodities for which Codex MRLs have not been set, draft MRLs should be set based on the submitted results of domestic and overseas supervised residue trials.

When the Committee sets MRLs for pesticides in livestock commodities, they determine the appropriate residue definition and prepare draft MRLs based on livestock feed studies. They consider the draft MRLs and dietary burdens set by the MAFF for livestock feed (see Chapter 2). In addition, no internationally agreed upon methods for setting MRLs for fish and shellfish have been established, but draft MRLs are prepared for pesticides that are directly treated in or used near inland water such as paddy fields, and are expected to remain in fish and shellfish based on data from supervised residue trials, monitoring data for agricultural chemical residues, predicted environmental concentrations (PECs) of agricultural chemicals in surface water areas, and bioconcentration factors (BCFs) (see Chapter 3).

Furthermore, with regard to the MRLs in honey, in addition to setting default values, draft MRLs are drawn up based on information on analysis methods, monitoring data, etc. (see Chapter 4).

Regarding pesticides that had been used in the past but are currently detected as contaminants, the extraneous MRLs (EMRLs) are set by monitoring agricultural chemical residues (see Chapter 5). In addition, when setting the MRLs for extremely low-level pesticides, in principle, the same regulation value as the Japanese default MRL (this is called the uniform limit in Japan) of 0.01 mg/kg shall be set (see Chapter 6). The commodity groups and representative commodities in MRLs that are set based on international harmonization are discussed in Chapter 7.

### 1-2. Concepts in the Preparation of Draft MRLs

#### 1-2-1. Determination of Definitions for Regulated Residues

Definitions for regulated residues in terms of setting and testing compliance with MRLs are determined based on the following basic requirements: 1) if possible, a single compound should be used to determine conformity to MRLs easily and quickly and at a reasonable cost; 2) the compound should be the most suitable one for the purpose of confirming compliance with Good Agricultural Practice (GAP); 3) the same definition should be used for all commodities as much as possible; and 4) the use of common metabolites and degradation products derived from multiple pesticides should be avoided as much as possible.

In determining regulated substances, the following information and data (e.g., plant metabolism, farm animal metabolism, environmental dynamics, the results of and analytical methods used in supervised residue trials) should be used:

1) The residue composition found in plant and animal metabolism studies

2) The nature of residues determined in supervised residue trials

3) The practicality of analytical methods used for regulatory purposes

4) Whether metabolites or analytes common to other pesticides are formed

5) Registration of use of the metabolite of the pesticide as another pesticide

6) The definitions of residues already established by national governments as well as long-used and customary accepted definitions

7) Lipophilicity

8) The joint FAO/WHO Expert Committee on Food Additives definitions for marker residues that are already established for compounds that may be identified as pesticide residues in livestock commodities

9) The contribution of metabolites subject to oral exposure by consumers (for risk assessment)

#### 1-2-2. Determination of Critical GAP (cGAP)

cGAP is a method recommended to register the assumed MRL (in principle, the maximum application dosage per unit area or use at the maximum treatment concentration, maximum number of treatments, or minimum PHI). In principle, to estimate MRLs, only data from supervised residue trials conducted based on cGAP are considered.

A certain number of independent supervised residue trials conducted based on cGAP are required. Supervised residue trials should be conducted according to a well-planned protocol in which differences in topography, cultivation/management methods, seasons, etc., are considered as a prerequisite for estimating reliable MRLs.

Unless there is a rational reason for data with identical GAPs to be obtained from an identical population based on a comparison using statistical methods, data from supervised residue trials conducted based on different GAPs should not be combined and evaluated. In the case that multiple residue levels are obtained in an identical trial with the conditions changed within the GAP range, a higher value should be selected (e.g., samples collected after PHI or with low application dosage). The practitioner is responsible for performing supervised residue trials in accordance with cGAP. The details of cGAP are shown as follows:

##### 1) Application dosage or treatment concentration (the active ingredient of the pesticide is applied over a specific area or per unit volume of an environmental component (e.g., air, water, soil))

In the case that the actual application dosage (or treatment concentration) in the supervised residue trial is within ±25% of the maximum application dosage in GAP and the other conditions are the same as in those in cGAP, the data from the supervised residue trial are used for setting MRLs.

In the case that the application dosage differs by 25% or more, but the others are implemented in accordance with cGAP, the principle of proportionality is applied. The principle of proportionality is applied to data obtained from supervised residue trials implemented within a ratio ranging from 0.3 to 4 times the GAP ratio or concentration, and the residue level of the trial is adjusted to the equivalent of cGAP by proportional calculation.

Adjustments in accordance with the principle of proportionality will be applied to the application dosage or concentration only, and not to other parameters. The principle of proportionality cannot be used for post-harvest situations. It is also recommended that the concept not be applied to hydroponic situations because of a lack of data.

##### 2) PHI

The degree of allowable change of acceptable intervals around PHI is determined by the ratio of decline of the residues of the compound under evaluation. In the case that it is reasonable based on the decline ratio, the range of PHI in which the residue level is ±25% can be judged to be within the range of cGAP. In the case that the decomposition is particularly rapid, this will be determined on a case-by-case basis.

##### 3) Number of treatments

When comparing the number of treatments in a supervised residue trial with the registered number of treatments, it is necessary to consider both the persistence of the compound and the interval between applications. Considering pesticides, the contribution to previous applications treated more than three half-lives (three times the half-life) prior to the final treatment is not considered to be significant.

##### 4) Formulations

In many trials, the differences in formulations would not affect the fluctuation in residue concentration compared with other factors. Even if the formulation is different, in the case that the usage is identical (e.g., dilute with water before use), in principle, the results from supervised residue trials can be replaced according to OECD guidelines (e.g., emulsifiable concentrates [ECs], wettable powders, water dispersible granules, flowable or suspension concentrates, soluble concentrates).

#### 1-2-3. Preparation of Draft MRLs Based on Data from Supervised Residue Trials

Based on the results of pesticide treatment according to cGAP in properly implemented supervised residue trials, the Committee should quantify the expected residue level range in commodities, determine the declining ratio of pesticide residues, and determine the supervised trial median residue (STMR) and highest residue (HR) values for exposure assessment before setting draft MRLs.

**Fig. 1:**
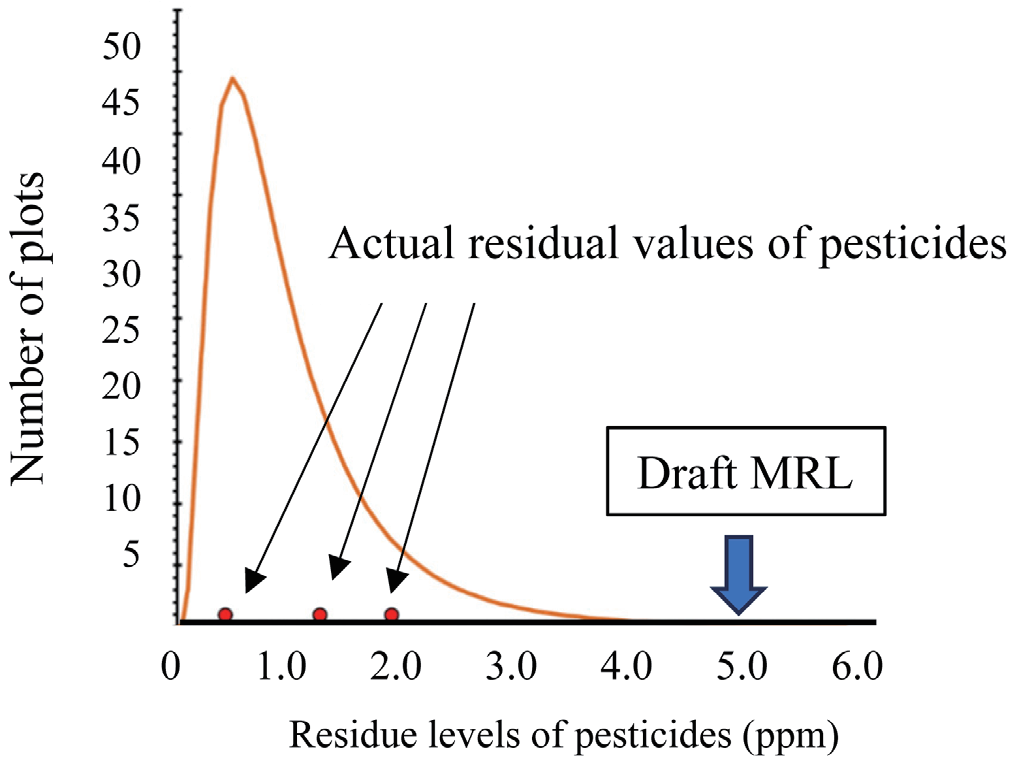
Frequency distribution of crop residue levels of pesticides Red dots indicate actual residual values of pesticides. Draft MRLs (arrow) are set by taking into account analytical errors.

The residue level of pesticides in commodities is known to fluctuate because of factors such as 1) standards for the use of pesticides, 2) cultivation conditions, 3) climate, and 4) varieties described in section 1-1, as shown in Fig. 1, when preparing draft MRLs based on data from supervised residue trials; in addition to these residual fluctuations, draft MRLs are set by taking analytical errors into account.

In the JMPR, the OECD MRL Calculator^3^^)^ was used as a statistical calculation method to estimate MRLs. The maximum value of the following three results calculated from supervised residue trial data would be set as a draft MRL: 1) the HR value (the HR value in supervised residue trials, which guarantees that the draft MRL will always be above the HR), 2) the arithmetic mean + 4 × standard deviation (the basic proposal value using the arithmetic mean and standard deviation of the data set), and 3) the 3 × arithmetic mean × correction factor [CF] method, in which the CF assures that the relative standard deviation of the data set is at least 0.5 concordant with the distribution of residues in data sets selected for the estimation of MRLs).

As the estimation of the draft MRLs by the OECD MRL Calculator requires statistical calculation, the number of data included in the data set must be 3 or more (In the case that the number of data is 3 to 7, a message indicating high uncertainty is displayed). Therefore, to use the OECD MRL Calculator, it is necessary to have at least three trials (preferably eight or more supervised residue trials; note that it is not necessary to conduct a supervised residue trial for each formulation with the same usage method).

Regarding the number of supervised residue trials required for pesticide registration in Japan, in principle, it is necessary to submit the results of six or more for major crops and their consumption levels, such as rice. In addition, for the purpose of increasing the number of trial results that can be used for setting MRLs, a commodity classification has been introduced, and the setting of MRLs for this group has been studied (see Chapter 7 for new commodity classifications, representative commodities, and the required number of supervised residue trials).

For commodities with a low production amount, from the viewpoint of securing results from three or more trials, it is recommended to use the principle of proportionality to implement residue trials for representative commodities assuming MRLs in a group, and to use trial results implemented overseas. After making efforts to secure the required number of supervised residue trials as described above, from the viewpoint of international harmonization, in Japan, in principle, the MRLs should be set using the OECD MRL Calculator.

However, for commodities with a low production amount and a small number of supervised residue trials, in addition to examining the use of the OECD MRL Calculator in three or more trials, in principle, MRLs should be set using the maximum value obtained in the supervised residue trial.

### 1-3. Concept for Accepting Trial Results Conducted Overseas for Crops Registered in Japan

Applying the principle of using data from supervised residue trials developed by the JMPR on a global scale, the following will be implemented for trials on crops grown on fields.

In the first step, for pesticides registered in Japan, in the case that a sufficient number of supervised residue trials that reflect cGAP in Japan are obtained (to establish import tolerance, cGAP of the country or region should be reflected), the data set is used for residue level estimation. For supervised residue trials conducted under conditions in which only the application dosage differs, residue levels are adjusted according to the principle of proportionality.

In the case that sufficient residue data are not obtained in the first step, residue data that conform to cGAP of other countries or regions (the same cGAP as already mentioned) or residue data adjusted by the principle of proportionality with cGAP will be considered together with the data obtained in the first step.

Data sets obtained in the first and second steps can be combined if they belong to the same statistical population (based on the Mann–Whitney *U* test or Kruskal–Wallis *H* test). However, if the data sets cannot be combined, which data set to use should be carefully considered.

In Japan, from the viewpoint of securing the required number of supervised residue trials necessary for setting MRLs, the JMPR concept mentioned before is introduced. In the case that the number of domestic supervised residue trials conforming to cGAP is fewer than the number of examples (or trials) for setting MRLs, supervised residue trials conducted overseas (conforming to cGAP in Japan) are accepted.

In the case that supervised residue trials conducted overseas do not comply with cGAP, residue data may be adjusted based on the principle of proportionality, if possible. However, in principle, all studies must conform to Good Laboratory Practice. In the case of registration for crops grown facilities, such as in greenhouses, trials conducted overseas are also accepted for setting MRLs when conducted in accordance with cGAP in Japan.

### 1-4. Estimation of Long- and Short-Term Dietary Intake

The estimation of long- and short-term dietary intake is conducted to confirm that there are no adverse health effects on humans with each draft MRL prepared based on the results from supervised residue trials and Codex MRLs. In conducting the dietary intake estimation, the target substances (i.e., those subjected to dietary intake estimation) are determined.

#### 1-4-1. Determination of Residue Definitions for Dietary Intake Estimation

Residue definitions for dietary intake are not necessarily identical to those of regulated residues. The targets are often different because they must include the metabolites and degradation products with toxicological concerns. For the determination, it is necessary to examine factors such as plant metabolism, animal metabolism, toxicity, crop residue, and changes due to processing, according to the literature.

#### 1-4-2. Long-Term Dietary Intake Estimation

Regarding long-term dietary intake, the amount of exposure to pesticides is estimated based on the sum of the draft MRLs or the mean values of supervised residue trials multiplied by the average intake of each commodity (according to a special tabulation report on commodity intake frequency and intake surveys for FY2005–2007), and the exposure level is confirmed to be within the ADI range for each classification of the entire population, including young children aged 1 year and older, those aged 1–6 years, pregnant women, and older adults (aged 65 years or older).

In the case that the estimated dietary intake exceeds the ADI range, after considering further refinement of the calculation, the Committee requests a study to change the GAP as necessary or examine a draft MRL based on the new GAP, and considers registering the corresponding pesticide as an applicable crop or deleting the draft MRL.

#### 1-4-3. Short-Term Dietary Intake

The short-term dietary intake is estimated based on the draft MRL for each commodity, the HR or median (STMR) values in the supervised residue trials, and the maximum daily intake of each commodity (based on the results of the 97.5th percentile for commodity intake, commodity intake frequency, and intake survey for FY2005–2007, and MHLW Science Research Report for FY2007–2012), and the intake is confirmed not to exceed the ARfD.

In principle, the Committee estimates the short-term dietary intake of each pesticide using HR values when the number of samples in supervised residue trials is four or more, and using the draft MRL when the number is three or fewer. In the case that the estimated short-term dietary pesticide intake exceeds the ARfD, the Committee considers pesticides individually in regard to requesting a GAP change, and reduces the level in the draft MRL after adding necessary data.

### 1-5. Understanding the Actual Dietary Intake for Consumers

Concerning the actual dietary intake for consumers through the eating and drinking of pesticides for which MRLs are set, this is trying to be understood by monitoring pesticide residue levels at quarantine stations, prefectures, etc., as well as through daily intake surveys using the market basket survey method.

It has been confirmed in surveys to date that the pesticide residue levels in agricultural commodities in circulation are low and do not cause problems in terms of commodity intake for consumers.

## 2. Preparation of Draft MRLs for Pesticides in Livestock Commodities

Residue levels in livestock commodities arise from the consumption of feed items containing pesticide residues or from direct application of a pesticide to livestock to control bacteria such as ectoparasites. When the MRL recommendations from two sources do not agree, the higher recommendation is adopted. Here, we describe only the case of consumption of feeding items containing residues.

**Fig. 2:**
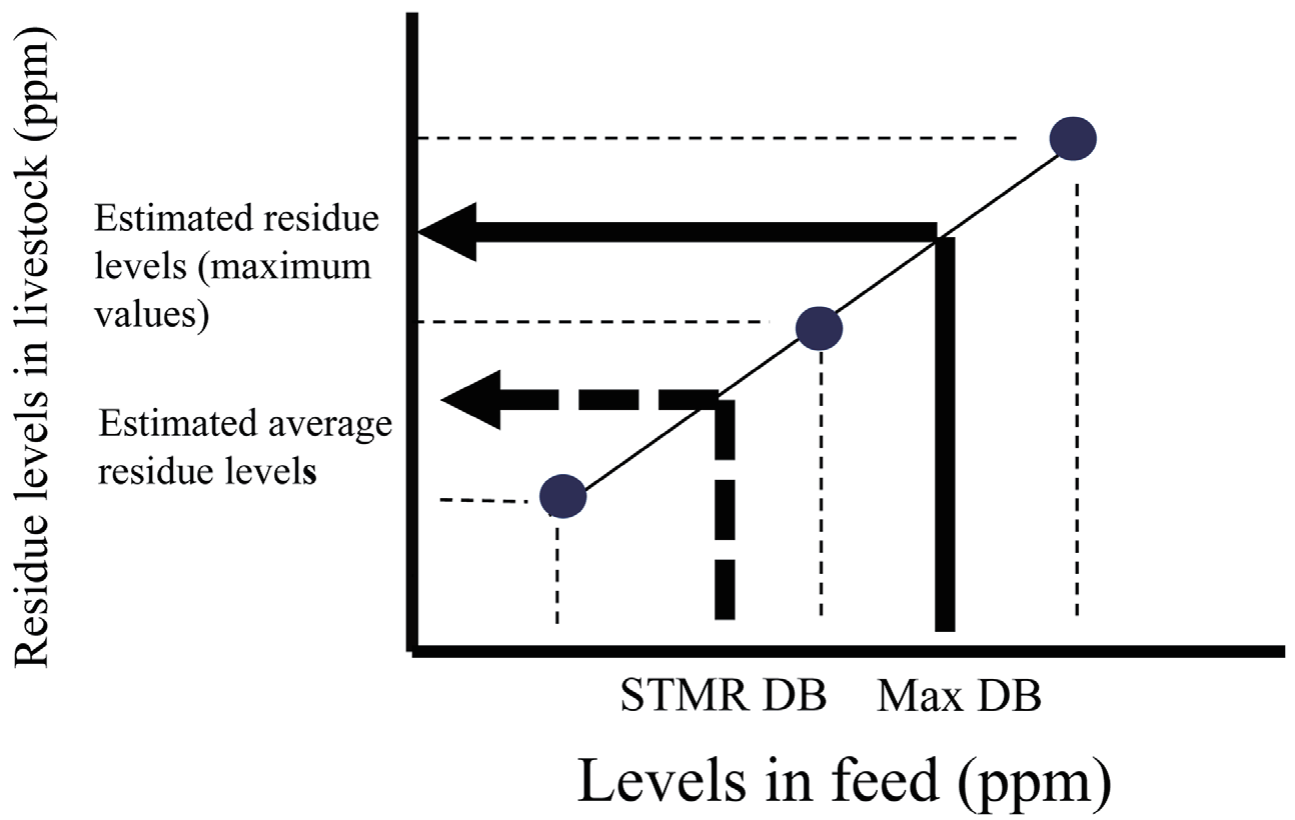
Estimation of residue levels (maximum values) and average residue levels in livestock commodities Estimated residue levels (maximum values) and estimated average residue levels in livestock commodities are obtained by interpolating the maximum dietary burden (MaxDB) and supervised trial median residue dietary burden (STMR DB) into the linear regression line obtained by the least-squares method using dosages (three levels) in livestock feeding studies (livestock residue trials), respectively. Solid arrow indicates the interpolation from MaxDB. Dashed arrow indicates the interpolation form STMR DB. Dots indicate the residue levels determined by three dosages in livestock feeding studies.

Estimated residue levels (maximum values) in livestock commodities (e.g., tissues, milk, eggs) are obtained by interpolating the maximum dietary burden (MaxDB) into the linear regression line obtained by the least-squares method using dosages (three levels) in livestock feeding studies (livestock residue trials), as shown in Fig. 2. In the case that the residue level does not fit a regression line, it is interpolated between two dosages containing the MaxDB.

In the case that the MaxDB is below the minimum dose in the feeding study and the straight line passes through the origin, the transfer factor (residue level of livestock commodities/applied dosage) is used to calculate the estimated residue level (maximum value) in livestock commodities by the MaxDB × the transfer factor. In the case that it does not pass through the origin, it will interpolate between the lowest dose and the control. In the case that the residue level of the lowest dose of the livestock commodity has not been measured, the result at the next dose for which the measurement was obtained is used. However, in the case that the residue level is below the limit of quantification (LOQ), even at doses much higher than the MaxDB, the MRL is determined as the LOQ.

In the case that the MaxDB exceeds the maximum dose in the feeding study but is within +30%, and there is linearity to that level, the MRL is determined by extrapolating the MaxDB above the linear regression line. In the case that there is no linearity, extrapolation is carried out using the residue level at the highest and second highest doses.

In the case that beef and dairy cattle have different MaxDB values, the larger value is used to calculate the estimated residue level (maximum value) for muscle, fat, liver, and kidney. Alternatively, all the estimated residue levels (maximum values) are calculated from each MaxDB of beef and dairy cattle, and the results are compared to select the maximum value. In addition, when excretion into milk is significant, the MRL of the visceral organs is set taking this into consideration.

In calculating the estimated residue levels (maximum values) of muscle, fat, liver, kidney, and eggs, the maximum individual residue level in each treatment group is used. However, for milk, the average value of each group in the steady state is used.

The estimated residue levels in livestock commodities (STMR or average residue levels) are obtained using the same procedure as the above calculation of the maximum value using the STMR dietary burden or the average feeding dose (average residue level in feed) instead of the MaxDB (Fig. 2) and using the mean residue level in the livestock in the relevant feeding group instead of the maximum individual residue level in the relevant feeding group. However, in many trials, information on the STMR dietary burden (average residue level in feed) cannot be obtained, so the MaxDB is used in such cases.

## 3. Preparation of Draft MRLs for Pesticides in Fish and Shellfish

### 3-1. Outline

Although the pesticides were not used for fish and shellfish, in the case that monitoring data for pesticide residues in fish and shellfish are available, the Committee can use these data to set MRLs. In the case that there is no use for the fish and shellfish and no monitoring data are available for the pesticide residues, the estimated residue level in fish and shellfish is calculated from the PEC and BCF, and the MRL is set from the estimated residue level.

Among the PECs, that in water area (water area PEC) is stipulated in the setting of pesticide registration standards to prevent damage to the livestock and plants in the environment based on Article 4, Paragraph 1, Item 8 of the Japanese Agricultural Chemicals Regulation Act. Compliance is carried out using the following methods from 1) to 3):

### 3-2. Basic Concept of PEC

Calculated by dividing the estimated inflow into the river due to surface runoff and spray drift by river flow.

#### 3-2-1. Pesticides Used in Paddy Fields

The water area PECs of pesticides used in paddy fields are estimated using the following tier system: 1) First stage (paddy field PEC tier 1): The Committee calculates this assuming that the entire amount of pesticide is dissolved in the paddy field water and not affected by decomposition, adsorption to soil and bottom sediment, etc., and flows into the river at a predetermined runoff ratio. 2) Second stage (paddy field PEC tier 2): The Committee calculates this considering the decomposition, adsorption to soil and bottom sediment of the pesticides in paddy fields and rivers, and water holding times, etc. 3) Third stage (paddy field PEC tier 3): The calculation formula is the same as that for paddy field PEC tier 2, but the study results in the actual paddy field are used as the parameter.

#### 3-2-2. Pesticides Used in Upland Fields (Non-Paddy Fields)

The water area PECs of pesticides used in upland fields are estimated using the following tier system: 1) First stage (upland field PEC tier 1): The Committee calculates this by regarding the pesticide as flowing into the river at a predetermined surface runoff and drift ratio. 2) Second stage (upland field PEC tier 2): The Committee calculates this by regarding the pesticide as flowing into the river at the surface runoff and drift ratio obtained by field studies, etc.

### 3-3. Estimated Residue Level in Fish and Shellfish

In examining the estimated residue levels in fish and shellfish, paddy field PEC tier 2 or upland field PEC tier 1 should be used. For residue pesticides used in both paddy and upland fields, the higher one is used. For the BCF, in principle, it is appropriate to adopt measured data obtained from some aquatic organisms, but when there are no measured data, the BCF is calculated from the value of log_10_Pow (Pow: octanol/water partition coefficient) by a relational expression (log_10_BCF = 0.80 × log_10_Pow – 0.52). The estimated residue level of fish and shellfish is obtained using the following formula: Water area PEC × BCF × 5.

Fish and shellfish should be distinguished from each other because of their different habitat, etc., for setting MRLs, but as there is a lack of knowledge at present about BCF calculations for shellfish, in principle, the MRL for shellfish is set using the BCF for fish.

## 4. Technical Guidelines for Setting MRLs for Pesticides, etc., in Honey

### 4-1. Outline

When honeybees collect nectar and pollen, they may be exposed to pesticides, etc., directly or indirectly, and thus, pesticides may be present in honey. The European Commission has published guidelines^4^^)^ for determining the magnitude of pesticide residue in honey and setting appropriate MRLs for honey to set safe thresholds for consumers.

Honey is a food of livestock origin, and as a general rule, there are three ways in which domestic honeybees are exposed to pesticides: 1) Exposure by direct application of chemicals. 2) Exposure as a result of the treatment of accommodations. 3) Exposure through nectar and pollen contaminated with pesticides.

In the cases of 1) and 2), the MRLs are set in consideration of residues owing to their use as veterinary drugs and in the treatment of beehives. In the case of 3), honeybees collect and ingest nectar and pollen from the crops treated with pesticides and the non-target plants growing in the vicinity are in bloom.

### 4-2. Basic Approach to Setting the MRLs

#### 4-2-1. Definition of Residue Substances

The definition of residue substances for enforcement in honey shall be the same as that in agricultural products. However, when determining the definition of residue substances, several points should be considered in addition to the basic principles indicated in the OECD guidelines. The points that should be considered are as follows: 1) Is the definition of substances for enforcement in crops, etc., appropriate?; 2) Do the official analytical methods, etc., used for enforcement cover the substances included in the definition of residue in honey?; and 3) Are analytical standards available for all components of the proposed enforcement definition of residue substances?

#### 4-2-2. How to Set the Draft MRLs

A default value of 0.05 ppm is set for pesticides used for major food crops that produce nectar (e.g., fruits, nuts). However, in the case that the estimated dietary intake of the pesticide, etc., exceeds the range of the ADI or ARfD as a result of the exposure assessment, the LOQs of the published test method that can measure lower concentrations are set.

In addition to syrup feeding trials, it is possible to set MRLs based on specific data such as monitoring data.

When setting MRLs based on monitoring data, the “As Low As Reasonably Achievable” (ALARA) principle is applied based on the same concept as that used to set MRLs for contaminants in food using statistical methods.

However, no internationally agreed upon value for the percentage of violation allowed in this case has been established, and is therefore left to the discretion of the risk management organization. In setting MRLs, they must ensure an adequate supply of food to consumers and not be an unreasonable restriction on trade. Based on the ALARA principle, and taking the international context into account, an acceptable violation ratio is set. The draft MRLs shall be set based on the percentile value of the relevant violation ratio in data that include those below the LOQ.

### 4-3. Dietary Risk Assessment

In Japan, the average daily intake of honey is 0.773 g/person/day, 0.471 g/person/day for young children, 1.127 g/person/day for pregnant women, and 1.058 g/person/day for older adults. This is a very small proportion of the total diet.

As a result, honey intake is not considered to have a significant effect on long-term dietary exposure assessments.

When setting an MRL based on IV-2-2, the intake amount from the relevant food is calculated using the LOQ or 0.05 ppm, but in general, the TMDI calculation results in an overestimation of exposure.

In the case of setting an MRL based on the monitoring data, the intake from the food may be set to 0 in cases where all the pesticide residue inspection data, etc., are below the LOQ, but in the evaluation of environmental contaminants, half of the LOQ may be used.

For this reason, in principle, dietary risk assessments shall be conducted using the numerical values as follows: 1) In cases where the substance is detected in 40% or more of the samples in tests for pesticide residues, etc., the median value of all data, including data below the LOQ of the analytical method, shall be used. 2) In cases where the residual level in 80% or more of the samples is less than the LOQ, the intake dose shall be set to 0. 3) In cases where the residual level at 60% or more but less than 80% of the samples is less than the LOQ, half of the LOQ shall be used.

However, dietary risk assessments are not limited to these values. An appropriate numerical value should be selected based on the characteristics of the pesticide to be assessed to the extent that the health of consumers can be protected, so as not to result in an excessive dietary risk assessment.

### 4-4. Review of the MRLs

Because it is expected that the levels of pesticides in honey will vary greatly depending on the region of the exporting country, etc., inspection data for residue levels of pesticides, etc., of food commodities from different exporting countries will continue to be collected, and the MRLs will be reviewed as necessary through the development and evaluation of official analytical methods.

(Reference) Main food crops that produce nectar include buckwheat, chrysanthemums, edible leaves, leeks, Chinese chives, tangerines, summer oranges, oranges (including navel oranges), grapefruit, lemons, limes, other citrus fruits, sunflowers, rape seeds, chestnuts, pecans, almonds, walnuts, coffee, other nuts, apples, Japanese pears, Western pears, quinces, loquats, peaches, apricots, plums, cherries, grapes, persimmons, strawberries, raspberries, blackberries, blueberries, huckleberries, cranberries, other berries, bananas, kiwis, papayas, avocados, pineapples, guavas, mangoes, passion fruit, dates, and other fruits.

## 5. Methods of Setting Standards for Chemical Substances Used as Pesticides in the past and Now Detected as Contaminants^5^^)^

### 5-1. Outline

The registration of pesticides has been halted, and for chemical substances detected as contaminants, unlike the pesticides used under GAP, international EMRLs have been set using monitoring data. In Japan, an identical method will be used to set EMRLs.

In addition to setting MRLs based on the results of supervised residue trials, the Codex Committee on Pesticide Residues (CCPR) may also set EMRLs based on the monitoring data of distribution products for chemical substances remaining in agricultural crops of environmental origin such as chlorinated chemical substances (e.g., dichlorodiphenyltrichloroethane, aldrin, dieldrin, endrin)^*1^ and pesticides remaining in spices^*2^. When the CCPR examined the concept of setting EMRLs in 1998–1999, 2%–5% or 0.2%–0.4% was discussed as a violation ratio, but this has been left to the judgment of each country.

When setting EMRLs based on monitoring data, it is considered appropriate to use statistical methods, etc., by applying the ALARA principle based on the same concept as the preparation of EMRLs for contaminants in commodities. However, no internationally agreed upon value for the percentage of violation allowed in this case has been established, and is therefore left to the discretion of the risk management organization. In setting EMRLs, they must ensure an adequate supply of commodities to consumers and not be an unreasonable restriction on trade. In reference to such international trends, EMRLs in Japan are set as follows.

### 5-2. Basic Concept of Setting EMRLs

#### 5-2-1. Commodities for Which EMRLs Are Set

The Commodities for which EMRLs are as follows: 1) Commodities that have been repeatedly and continuously detected (detected commodities) through monitoring and voluntary inspections of imported and domestic commodities in Japan (inspection of residue pesticides), and 2) Commodities for which Codex EMRLs are set.

#### 5-2-2. Setting Method for Draft EMRLs

For detected commodities for which Codex EMRLs are not set, based on the ALARA principle, an acceptable violation ratio is set based on the international situation. At that time, a draft EMRL is set based on the percentile for the violation ratio for data including those below the lower LOQ (treated as containing the same level as the lower LOQ).

For commodities for which Codex EMRLs are set, they are set in principle. For commodities in which such substances are detected during inspection of residue pesticides, etc., the higher value will be used as the draft EMRL when set by the same concept as Codex EMRL.

In the case that Codex EMRLs are set for livestock commodities, generally for highly fat-soluble substances, the EMRL is set in the fatty part of the meat. In such cases, in principle, the EMRL is not set for muscle tissue, but rather, with reference to the Codex EMRL for fat only.

### 5-3. Dietary Intake

Measures have been taken for the removal of chemical substances that had been used as pesticides in the past and are now detected as contaminants. Therefore, the Committee considered that the TMDI calculation can overestimate dietary exposure. In addition, in commodities for which all monitoring data of residue pesticides, etc., are below the lower LOQ, the intake from the commodity may be set to 0, but in the assessment of environmental contaminants, half of the lower LOQ may be used.

For this reason, in principle, dietary risk would be assessed using the following values from 1) to 3). However, based on the characteristics of pesticides subject to dietary intake, appropriate values should also be selected so as not to overestimate exposure within the scope of protecting the health of consumers. These three values are as follows: 1) For commodities in which the chemical substance is detected in 40% or more of the sample via inspection of residue pesticides, etc., the median of all data, including data below the lower LOQ of the analytical method for the commodity, is used. 2) In the case that the residue level in 80% or more of the sample is below the lower LOQ, the intake from the commodity is set to 0. 3) For commodities in which the agricultural chemical residue in 60%–80% of the sample is below the lower LOQ, or the chemical substance is not detected via inspection of residue pesticides, etc., but for which the Codex EMRLs are set and adopted, the value is set to half the lower LOQ.

In the case that the value exceeds 80% of the tolerable daily intake (TDI), the EMRL shall not be set from commodities that have not been detected via the inspection of residue pesticides, etc., among commodities for which Codex MRLs have been set (regulation based on a uniform limit of 0.01 mg/kg). Even if all the EMRLs of commodities for which Codex EMRLs are set are deleted and still exceed 80% of the TDI, measures such as reducing the EMRLs of commodities detected via inspection of residual pesticides, etc., should be taken.

### 5-4. Review of EMRLs

Because the level of the chemical substance is assumed to vary greatly depending on the commodity and regional differences in the exporting country, we will continue to collect data on the monitoring of residue pesticides, etc., for commodities of different exporting countries, and to conduct market basket surveys and revise the EMRLs as necessary.

## 6. Concept of Setting MRLs for Extremely Low-Level Pesticides^6^^)^

### 6-1 Outline

Regarding MRLs for pesticides in commodities, even if the results from supervised residue trials that are the basis for setting the MRLs are below the lower LOQ, considering the usage and number of trials, etc., the MRLs are set in consideration of natural variations in the results of residue trials so that crops that used pesticides properly do not violate the Japanese Food Sanitation Act. However, using this method, an MRL higher than necessary may be set for an extremely low-level pesticide. In addition, the MRLs for some pesticides registered in Japan are not set if they are considered to no longer be remaining in commodities. However, to conduct appropriate dietary intake and residue monitoring studies in such cases, the MRLs are set as follows.

### 6-2 Setting of MRLs for Extremely Low-Level Pesticides

As pesticides that meet the following conditions 1) and 2) are considered to be hardly remaining, the Committee would like to use the lower LOQ as the MRL when setting MRLs: 1) As long as they are used appropriately, pesticides such as soil fumigants are considered to be very unlikely to remain. It should be noted that the possibility of remaining being extremely low can be explained rationally based on other study results. 2) In the case that supervised residue trials are conducted, all results should be below the lower LOQ.

In the case that a supervised residue trial with a lower LOQ of 0.01 ppm or less is being conducted, the same regulation value of 0.01 ppm as the Japanese default MRL will be set as the residue standard.

In addition, in the case that it is reasonably clear that the pesticide does not remain and is registered as a pesticide in Japan, even if no supervised residue trial is conducted, the same regulation value as the Japanese default MRL (0.01 ppm) will be set as the residue standard.

However, in cases where there are concerns about safety, such as when the MRL does not fall within the allowable range of the ADI, individual regulations should be considered on a case-by-case basis, such as by setting the MRL based on the same approach as before.

### 6-3 Safety Concept

In the case that the MRL should not be set higher than the actual residue level, and there is a registration for pesticides that are very unlikely to remain, by setting the same regulation value as the Japanese default MRL (0.01 ppm) as the residue standard, it will be subject to dietary intake and residue monitoring inspection, and thereby possible to set a more appropriate and practical MRL.

## 7. Commodity Groups and Representative Commodities in Setting MRLs Based on International Harmonization^7^^)^

Regarding the setting of MRLs for pesticides, the identical method with reference to the concept of internationally implemented group MRLs and the status of revision of commodity classifications by the CAC is used in Japan. The commodity groups that can be categorized by the same MRL and the representative commodities for which supervised residue trials necessary for setting the MRL are to be clarified, and international harmonization of setting group MRLs is planned^8^^,^^9^^)^.

Based on the results of science research conducted by the MHLW^10^^)^, the commodity group classifications and representative commodities selection for setting group MRLs in Japan were organized according to the following concepts.

### 7-1 Concept of Setting Commodity Groups and Representative Commodities Selection

#### 7-1-1 Commodity Group Classifications

Based on the commodity group classifications by the CAC, revisions should be made according to the practical use in Japan (e.g., dietary intake, size of vegetables). In addition to botanical classifications, consider dietary risk of pesticides and residue level of pesticides by portion and morphology. So-called minor commodities with low production are not subject to supervised residue trials, are low in terms of dietary intake, and make only a small contribution to health risks; therefore, they should be included in the group containing major or sub-major commodities as much as possible. Based on this concept, for commodities with different analytical portions despite being set in the same commodity group, changing the analytical portion should be considered.

#### 7-1-2 Representative Commodities Selection

With reference to the CAC guidelines^11^^)^, set representative commodities to undergo supervised residue trials in each commodity group (including major and medium classifications). The Committee considers domestic production when selecting representative commodities so that pesticide registration in Japan can be managed.

### 7-2. Commodity Groups and Representative Commodities

New commodity groups and representative commodities are set based on the concept described in 7-1 and shown in **Table 1**.

**Table 1-1.** Commodity groups and representative commodities

Commodity Group	Subgroup	Commodity	Required representative commodities and the number of supervised residue trials		
			Representative commodities for setting MRL of the subgroup		Representative commodities for setting MRL of the commodity group
Citrus fruits	Large sized citrus fruits	Grapefruit	Natsudaidai, Hassaku or Grapefruit: 3 trials(If the usage standard is the same as the medium size, the results of the medium size can be used)		Unshu orange or Orange: 6 trials; Lemon, Yuzu or other small sized Mandarins or Kumquats: 3 trials
		Natsudaidai			
		Other large sized citrus fruits			
	Medium sized citrus fruits	Unshu-orange	Unshu orange or Orange: 6 trials		
		Oranges (including Navel Orange)			
		Other medium sized citrus fruits			
	Small sized citrus fruits	Lemon	Lemon, Yuzu other small sized oranges or Kumquats: 3 trials		
		Lime			
		Other small sized citrus fruits			
Pome fruits	Rosaceae pome fruits (including Persimmons)	Apple	Apple and Pear: 12 trials in total (4 or more trials for one type of crop)		
		Persimmon, Japanese			
		Nashi pear			
		Pear			
		Loquat			
		Quince			
		Other pome fruits			
Stone fruits	Outou (Cherries)	Outou (including Cherries)	(Set by the commodity)		
	Plums	Japnese plum (including Prunes)	(Set by the commodity)		
	Peaches	Peach	Peach or Ume: 3 trials		
		Ume (Japanese apricot)			
		Anzu (including Apricot)			
		Nectarine			
Berries and small fruits	Rose berries (excluding rose nuts)	Blackberry	Blackberry or Raspberry: 3 trials	Blueberry or Currants: 3 trials Grapse: 3 trials	Blueberry or Currants: 3 trialsStrawberry: 3 trialsGrapes: 3 trials
		Raspberry			
		Other rose berries			
	Azalea and Currant berries (shrubs) and rose fruits	Blueberry	Blueberry or Currants: 3 trials		
		Huckleberry			
		Cranberriy			
		Other Azalea and Currant berries			
	Other berries	Other berries	(Set by the commodity)		
	Grapes	Grapes	(Set by the commodity)		
	Strawberries	Strawberry	(Set by the commodity)		

**Table 1-2.** continued

Commodity Group	Subgroup	Commodity	Required representative commodities and the number of supervised residue trials	
			Representative commodities for setting MRL of the subgroup	Representative commodities for setting MRL of the commodity group
Tropical fruits (edible peel)	Tropical fruits(edible peel)	Guava	Guava or Fig or Olives: 3 trials	
		Date (Jujube)		
		Other tropical fruits (edible peel)		
Tropical fruit (not edible peeel)	Tropical fruit(not edible peel)	Banana	Kiwi fruits: 3 trialsBanana: 3 trialsPineapple: 3 trials	
		Kiwi fruit		
		Pineapple		
		Avocado		
		Mango		
		Papaya		
		Passionfruit		
		Other tropical fruits (not edible peel)		
Bulb vegetables	Onions (including bulb crops and lily roots)	Onion, Bulb	Onion or Garlic: 6 trials	Onion: 6 trialsWelsh onion or Chinese chive: 6 trials
		Garlic		
		Other onions		
	Green onions	Welsh onion (including leek)	Welsh onion, Chives, Onion, Beltsville bunching or Chinese chives: 6 trials	
		Chinese chives		
		Onion, Beltsville bunching		
		Other green onions		
Brassica vegetables (excluding Brassica leafy vegetables)	Flower buds	Broccoli	Broccoli: 3 trials	Broccoli: 3 trials Kohlrabi or Stem mustard: 3 trials
		Cauliflower		
		Other brassica flower buds		
	Stem vegetables	Oily family stem vegetables	Kohlrabi or Stem mustard: 3 trials	
Cucurbitaceous vegetables	Immature cucurbitaceous vegetables (those harvested immature)	Cucumber (including Gherkin)	Cucumber: 6 trials Zucchini: 3 trials	Cucumber: 6 trialsZucchini: 3 trialsPumpkins or Melons: 3 trials
		Melon, Oriental Picking		
		Other immature cucurbitaceous vegetables		
	Mature cucurbitaceous vegetables (those that are harvested after they mature)	Pumpkins (including Squash)	Pumpkins or Melons: 3 trials	

**Table 1-3.** continued

Commodity Group	Subgroup	Commodity	Required representative commodities and the number of supervised residue trials	
			Representative commodities for setting MRL of the subgroup	Representative commodities for setting MRL of the commodity group
Fruits and vegetables other than cucurbits	Tomatoes	Tomato	Tomato and cherry tomato: 6 trials in total (3 or more cherry tomato)	Tomato and cherry tomato: 6 trials in total (3 or more cherry tomato)Sweet peppers: 3 trials Chili peppers: 3 trialsOkra: 3 trialsEggplant: 6 trials
		Other tomatoes		
	Sweet pepper Chili pepper (including Okra)	Sweet peppers	Sweet peppers: 3 trials Chili peppers: 3 trials Okra: 3 trials	
		Okra		
		Other sweet peppers and peppers		
	Eggplants	Eggplant	(Set by the commodity)	
Leafy vegetables (including Brassica leafy vegetables)	Brassical leafy vegetable	Cabbage	Cabbage or Chinese cabbage: 6 trials Komatsuna, Mizuna or Radish leaves: 3 trials	Cabbage or Hakusai: 6 trialsKomatsuna, Mizuna or Radish leaves: 3 trialsLettuce and non-head lettuce: 6 trials in total (4 or more non-head lettuce trials)Spinach: 6 trials
		Brussels sprouts		
		Hakusai (Chinese cabbage)		
		Komatsuna		
		Mizuna		
		Chinese cabbage (type pak-choi)		
		Radish leaves (including radish)		
		Turnip leaves		
		Kale		
		Watercress		
		Other cruciferous leafy vegetables		
	Leafy vegetables of Composite family	Lettuce (including salad vegetables and Chisha)	Lettuce and non head-forming lettuce: eight trials (non-head-forming lettuce: more than 4 trials)	
		Endive		
		Chicory		
		Syungiku		
		Other leaf vegetables of the Composite family		
	Leafy vegetable of Amaranthaceous family	Spinach	Spinach: 6 trials	
		Other leafy vegetables of		
		Amaranthaceous family		
	Leafy vegetables of Umbelliferae family	Mitsuba	Mitsuba Parsley or Coriander: 3 trials	
		Parsley		
		Other leafy vegetables of Umbelliferae family		
	Other leafy vegetables	Other leafy vegetables (including baby leaves)	Perilla or other crops: 3 trials	
	Sprouts	Sprouts	Bean or Mung bean sprouts: 3 trials	

**Table 1-4.** continued

Commodity Group	Subgroup	Commodity	Required representative commodities and the number of supervised residue trials	
			Representative commodities for setting MRL of the subgroup	Representative commodities for setting MRL of the commodity group
Immature beans	Immature beans (edible pods and seeds)	Beans with pods	Beans with pods: 3 trialsSoya beans (succulent seeds): 3 trials	Beans with pods: 3 trialsSoya bean (succulent seeds): 3 trialsPeas or Broad bean: 3 trials
		Immature peas		
		Soya bean (succulent seeds)		
		Other immature beans (edible pods and seeds)		
	Immature beans (edible seeds)	Immature peas (green peas)	Peas or Broad bean: 3 trials	
		Other immature beans (edible seeds)		
Mature beans	Common bean/Cowpea	Red beans	Any one of crops: 3 trials	Soy bean: 6 trialsAny one crop of either Common beans or Cowpea: 3 trialsPeas or Broad bean: 3 trials
		Other common beans and		
		Cow peas		
	Soy bean	Soy bean	(Set by the commodity)	
	Peas	Peas	(Set by the commodity)	
	Peanuts	Peanuts	(Set by the commodity)	
	Other beans	Broad bean	Broad bean: 3 trials	
		Other beans		
Root vegetables	Potatoes	Potato	Potato or Sweet potato: 6 trials	Potato: 6 trialsRadish: 6 trialsCarrot: 6 trials
		Sweet potato		
		Taro (including Yasugashira)		
		Yams (Chinese yam)		
		Konjac		
		Other potatoes		
	Other root vegetables (excluding aqueous plants)	Roots of radish (including radish)	Radish: 6 trials Carrot: 6 trials Turnip: 3 trials	
		Sugar beet		
		Carrot		
		Parsnip		
		Burdock		
		Salsify		
		Turnip roots		
		Horseradish		
		Ginger		
		Other root vegetables (excluding aqueous plants)		
Root vegetables (Continued)	Roots, tubers, etc. of aqueous plants	Lotus root		
		Arrowhead		

**Table 1-5.** continued

Commodity Group	Subgroup	Commodity	Required representative commodities and the number of supervised residue trials	
			Representative commodities for setting MRL of the subgroup	Representative commodities for setting MRL of the commodity group
Stem vegetables	Stems and petioles	Celery	Celery: 3 trials	Celery: 3 trialsAsparagus: 3 trials
		Other stems and petioles		
	Stem and sprout vegetables	Asparagus	Asparagus: 3 trials	
		Bamboo shoots		
		Other stems and shoots		
	Other stem vegetables	Artichoke	Any one of crops: 3 trials	
		Other stem vegetables		
Edible Flowers	Edible Flowers	Edible Chrysanthemum	Edible Chrysanthemum or other edible flowers: 3 trials	
		Other edible flowers		
Mushrooms (cultivated)	Mushrooms	Shiitake mushroom	Shiitake mushroom: 3 trials Any one of other crops: 3 trials	
		Mushroom		
		Other mushrooms		

**Table 1-6.** continued

Commodity Group	Subgroup	Commodity	Required representative commodities and the number of supervised residue trials	
			Representative commodities for setting MRL of the subgroup	Representative commodities for setting MRL of the commodity group
Cereals (including pseudo cereals)	Wheat, Wheat-like grains with or without shells	Wheat	Wheat: 6 trials	Rice: 6 trialsBarley: 3 trialsCorns: 3 trialsImmature corns: 3 trials
		Rye		
		Other Wheat, Wheat-like grains and pseudo-grains without shells		
	Barley, Barley-like grains and pseudo cereals with shells or without shells	Barley	Barley: 3 trials	
		Buckwheat		
		Other barleys, barley-like grains and pseudo-grains with shells		
	Rice	Rice (Brown rice)	(Set by the commodity)	
	Sorghum and millet	Sorghum and millet	Any one of crops: 3 trials	
	Corns	Corns	Corns: 3 trials	
Gramineous crops for sugar and syrup production		Sugar cane	(Set by the commodity)	
Nuts (excluding peanuts)	Nuts (excluding peanuts)	Chestnut	(Set by the commodity)	
		Almond	(Set by the commodity)	
		Walnut	(Set by the commodity)	
		Pecan	(Set by the commodity)	
		Ginkgo	(Set by the commodity)	
		Other nuts	(Set by the commodity)	
Oil seeds	Oil seeds	Rape seed	Rapeseedor any one of other crops: 3 trials	
		Sesame seed		
		Safflower seed		
		Sunflower seed		
		Cotton seed		
		Other oil seeds		
Seeds for beverage production	Seeds for beverage production	Cacao bean	(Set by the commodity)	
		Coffee bean	(Set by the commodity)	
Tea	Tea	Tea	(Set by the commodity)	
Hop	Hop	Hop	(Set by the commodity)	
Herbs	Herbs	Herbs	(Set by the commodity)	
Spices	Spices	Spices	(Set by the commodity)	

In addition to conventional individual commodities, a new commodity classification is added to the small category based on the concept described in 7-1. The minor classification is set to include other individual commodities based on the results of the “Commodity Intake Frequency/Intake Survey” currently being conducted. A representative commodity is set for each major and medium classification.

### 7-3. MRL-Setting Method for Each Commodity Group

It is possible to set an MRL for each of the major and medium classifications by conducting more than the number of supervised residue trials shown with the representative crops within the same GAP range. However, the food intake required for the dietary risk assessment by the new major, medium, or minor classification will be aggregated by the “Food Intake Frequency/Intake Survey” currently underway; therefore, for the time being, for each minor classification belonging to a corresponding major or medium classification (e.g., citrus fruits), the same MRL shall be set for each classification.

When studies are required for multiple representative commodities, setting group MRLs is based on the premise that the degree of pesticide residue levels in the commodities under the major or medium classification is not significantly different. Referring to the JMPR concept^12^^)^, in the case that it cannot be derived from a statistically different population, the group MRL is set by the combined results of residue trials for each representative commodity.

When the Committee considers the group MRL to be derived from a statistically different population, the largest value obtained from the residue trial data of each representative commodity of the median of the supervised residue trial data for each representative commodity should be determined as the group MRL (it is inappropriate to evaluate the data together) under the condition that the ratio of the maximum to the minimum value is five times or less.

Although no revisions are made to the long- and short-term dietary intake methods, the summary value of the Food Intake Frequency/Intake Survey currently being conducted should be used for the dietary intake for each new major, medium, and minor classification.
